# Knowledge, Attitudes and Perceptions of Immigrant Parents Towards Human Papillomavirus (HPV) Vaccination: A Systematic Review

**DOI:** 10.3390/tropicalmed5020058

**Published:** 2020-04-09

**Authors:** Faeza Netfa, Mohamed Tashani, Robert Booy, Catherine King, Harunor Rashid, Susan R. Skinner

**Affiliations:** 1 The University of Sydney Children’s Hospital Westmead Clinical School, Discipline of Child and Adolescent Health, Sydney, NSW 2145, Australia; Toshani2003@Gmail.com (M.T.); robert.booy@health.nsw.gov.au (R.B.); catherine.king@health.nsw.gov.au (C.K.); harunor.rashid@health.nsw.gov.au (H.R.); rachel.skinner@health.nsw.gov.au (S.R.S.); 2 Kids Research, The Children’s Hospital at Westmead, Westmead, NSW 2145, Australia; 3 Faculty of Medicine, University of Tripoli, Ain Zara 13275, Libya; 4 National Centre for Immunisation Research and Surveillance, The Children’s Hospital at Westmead, Westmead, NSW 2145, Australia

**Keywords:** cervical cancer, human papillomavirus, HPV vaccine, knowledge, attitudes and perceptions

## Abstract

Background: Our understanding about knowledge, attitudes and perceptions (KAP) of immigrants regarding human papillomavirus (HPV) vaccine is poor. We present the first systematic review on KAP of immigrant parents towards HPV vaccine offered to their children. Methods: Major bio-medical databases (Medline, Embase, Scopus and PsycINFO) were searched using a combination of keyword and database-specific terms. Following identification of studies, data were extracted, checked for accuracy, and synthesised. Quality of the studies was assessed using the Newcastle Ottawa Scale and the Joanna Briggs Institute Qualitative Assessment tool. Results: A total of 311 titles were screened against eligibility criteria; after excluding 292 titles/full texts, 19 studies were included. The included studies contained data on 2206 adults. Participants’ knowledge was explored in 16 studies and ranged from none to limited knowledge. Attitudes about HPV vaccination were assessed in 13 studies and were mixed: four reported negative attitudes fearing it would encourage sexual activity; however, this attitude often changed once parents were given vaccine information. Perceptions were reported in 10 studies; most had misconceptions and concerns regarding HPV vaccination mostly influenced by cultural values. Conclusion: The knowledge of HPV-related diseases and its vaccine among immigrant parents in this study was generally low and often had negative attitude or perception. A well-designed HPV vaccine health educational program on safety and efficacy of HPV vaccination targeting immigrant parents is recommended.

## 1. Introduction

Human papillomavirus (HPV) infection is a sexually transmitted disease and both women and men are rapidly exposed to it after the onset of sexual intercourse [1,2]. Oncogenic HPV can cause cervical, anogenital, head and neck cancers [3,4].

Cervical cancer is the fourth most common cancer found in women and the third most frequent cause of death with approximately 570,000 cases and 311,000 deaths in 2018 worldwide [5,6]. In developed countries nearly half of the cervical cancer cases are diagnosed in women aged less than 50 years old [6,7]. Rates of HPV infection vary greatly between geographic regions and population groups. In developed countries, cervical cancer has been declining for many years largely due to the cervical cytology screening programme which is now being replaced by HPV screening. However, cervical cancer is increasing in developing countries where nationwide cervical cancer screening is currently unavailable. It is the second most common cancer in countries with a lower human development index ranking and is the most common cancer in about 28 countries [6,8]. The high-risk types, HPV 16 and HPV 18, cause 70% of all invasive cervical cancers and HPV types: 6, 11, 16, 18, 31, 33, 45, 52 and 58 together can cause 95% of cervical cancers.

HPV vaccination is the most effective method of preventing HPV infection [9]. The immunity gained via HPV vaccination is mainly responsible for the reduction in HPV infection and related cancers [10]. The main goal of this vaccination is to avoid persistent infections that may progress to an invasive carcinoma [10,11]. HPV vaccine is safe, well tolerated and has the potential to significantly reduce the incidence of HPV-associated precancerous lesions [12,13]. It can also effectively protect against certain HPV types that can lead to genital warts. This vaccine is most beneficial if delivered prior to the commencement of sexual activity [13,14]. During the last 12 years, over 80 countries have introduced national HPV vaccination programs [15]. The United States of America (USA), Australia, Canada and the United Kingdom (UK) were among the first countries to introduce HPV vaccine into their national immunization programs (Table 1). All countries programs target young adolescent girls, with some countries also having programs for adolescent males [16]. Specific target age groups differ as do catch-up vaccination recommendations. The majority of countries are delivering vaccine through school-based programs, health centres or primary care providers [15]. National HPV vaccination programs of two or three dose schedules have demonstrated a dramatic impact on population level HPV prevalence, persistent HPV infection, genital warts, and cervical intraepithelial neoplasia [17]. The coverage of HPV vaccine achieved by the national programs has been highly variable within the countries [13]. During the past ten years, since HPV vaccine was licensed, there has been an increase in immigrants from different cultures and languages travelling to the Western countries. Most of the immigrants originate from socio-economically underprivileged countries [17,18], and do not have a nationally funded HPV vaccination program (Table 1); therefore, it is reasonable to believe that most immigrants do not have a background knowledge about HPV vaccination.

Knowledge and understanding of HPV infection and HPV vaccine are important factors in decision-making about disseminating the vaccine [13]. Since the licensure of HPV vaccine in 2006, research regarding the uptake of HPV vaccine among ethnic minorities, immigrants and refugees, has been limited [18,19]. This is attributed to factors such as language barrier and cultural differences, legal issues, religion, education, lack of specialized migrant health services and lack of awareness among migrants of their rights [20]. To our knowledge, there is no systematic study on immigrant parents’ knowledge, attitudes and perceptions (KAP) towards HPV vaccination. This study aims to address this research gap by systematically synthesising published data on immigrant parents’ KAP towards HPV disease and vaccination offered to their children to inform future efforts to increase HPV vaccine coverage.

## 2. Materials and Methods

Literature searches were performed using OVID Medline (1946–April 2019), OVID Embase Classic (1947–April 2019), PsycINFO (1806–May 2019) and SCOPUS (1945–May 2019). The searches used a combination of data base-controlled vocabulary terms and text word terms. These included “Papillomavirus vaccines”, “Human Papillomavirus vaccine”, “knowledge, attitudes, perceptions”, “emigrants”, “immigrants”, “population groups”, “ethnic groups”, “refugees”, “mothers”, “fathers” and “parents”. Searches were conducted from 2007 to 2019. The final search was conducted on 1 May 2019. No language or date restrictions were applied. The OVID Medline search strategy used is available upon application to authors. We additionally searched the reference lists of review articles to identify original research articles describing knowledge, attitudes and perceptions of HPV vaccine among immigrant parents.

For inclusion in this review, papers needed to discuss knowledge or attitudes or perceptions of immigrant parents (defined as parents who have been permanently living in a foreign country along with their children) and/or primary immigrant caregivers towards HPV vaccine. Papers were excluded if they did not include the views of parents or only discussed other childhood vaccines. Perception was defined as how parents interpreted/perceived HPV vaccine in light of their life experiences, and attitude was defined as their reactions to those perceptions. After screening the titles, full texts were retrieved and reviewed, and data were extracted in an Excel sheet by the first author. The data collection form included the author, year, country of study, method, population, result of the study. Another author (HR) checked data abstraction and any discrepancy was resolved through discussion then data were synthesised. The quality of included studies was assessed by Newcastle Ottawa Scale (NOS) for assessing the quality of nonrandomised studies in meta-analyses http://www.ohri.ca/programs/clinical_epidemiology/oxford.asp and by Joanna Briggs Institute (JBI) Critical Appraisal tools for use in JBI Systematic Reviews Checklist for Qualitative Research https://joannabriggs.org/sites/default/files/2019-05/JBI_Critical_Appraisal-Checklist_for_Qualitative_Research2017_0.pdf.

## 3. Results

**Figure 1:**
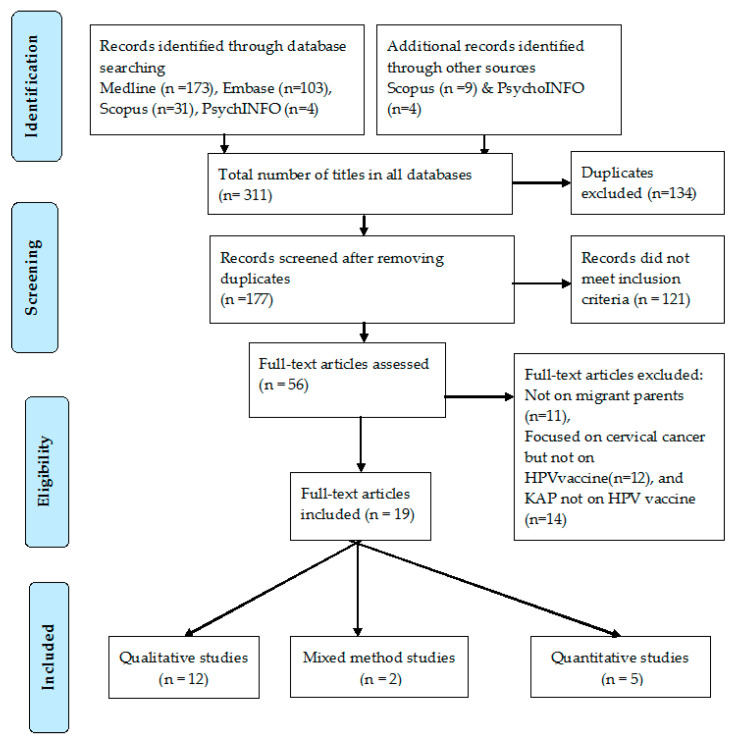
PRISMA flow diagram of the systematic review.

In this systematic review, 311 titles from four databases were retrieved in total. There were 134 duplicates leaving 177 records to be screened. Of 177 titles, 121 were excluded for not meeting inclusion criteria. The full texts of the remaining 56 titles were assessed. Of these 36 studies were determined to be out of scope of this systematic review and excluded with reasons, the remaining 19 articles met the eligibility criteria of the systematic review as shown in the PRISMA flowchart (Figure 1). There were 12 qualitative studies and five quantitative studies and two mixed method studies.

Total number of participants in all included studies was 2206 (M = 74, F = 1976 in addition to 156 parents with gender unclassified) with a male to female ratio of 1:27, where data were provided. Where age of interviewees was mentioned, the range varied from 18 to 66 years. Twelve studies were conducted in the USA, three in the UK, one in the Netherlands, one in Denmark, one in Sweden, and one in Puerto Rico. Six studies were conducted in community organizations including faith-based centres like churches and mosques [21,22,23,24,25,26], eight in health and social service agencies [27,28,29,30,31,32,33,34], two in schools and/or community groups [35,36], another two in social clubs [37,38], and one in a household [39].

Of the 19 studies, 16 reported on knowledge of the immigrant parents about HPV vaccine (Table 2), 13 reported their attitudes (Table 3) and 10 recorded perceptions (as defined by study author) towards HPV vaccine (Table 4). Four studies reported knowledge and attitudes [21,27,30,37] and one reported knowledge and perceptions [26], seven studies reported on all three outcomes (knowledge, attitude and perceptions) [22,23,29,35,36,38,39].

All included studies discussed the KAP of immigrant populations. If the study author(s) used the term “ethnic minority” to represent, we have similarly reported this term in the result tables.

For knowledge, the level of parents’ knowledge about HPV disease and HPV vaccine ranged from no knowledge in 11 studies [21,22,23,24,26,27,29,33,35,37,39] to limited knowledge regarding HPV and HPV vaccine, as they heard about the vaccine but they did not know HPV vaccine’s purpose, the eligibility requirements for the vaccine, and the vaccine’s dosing/schedule requirements in three studies. Five studies revealed that some participants had not heard of HPV disease or HPV vaccine [27,33,35,39]. There were four studies that reported participants had no prior knowledge of HPV as a sexually transmitted disease or as a cause of cancer [25,30,32]. In four studies, participants described a lack of information and knowledge about the purpose of HPV vaccination, and HPV transmission [21,29,37]. Two studies found participants had limited knowledge regarding the relation between sexual transmission of HPV and cervical cancer [22,36] (Table 2).

In regards to attitudes towards HPV disease and HPV vaccine (Table 3), a number of non-vaccinating ethnic minority parents had negative attitudes to HPV vaccination thinking it would encourage unsafe sexual practices and promiscuity [22,30,35]. However, three studies showed that once parents were informed about the vaccine during the focus groups, they became keen to vaccinate their children [34,36,37]. Non-vaccinating and partially vaccinating parents from various ethnic backgrounds expressed concerns about potential side effects [35]; religious values and cultural norms also influenced vaccine decision-making [28,29], and a majority of participants (regardless of vaccination status) had a more positive attitude towards vaccination when they received information about HPV vaccine (Table 3).

Participants had misperceptions about HPV vaccine. The main reasons for declining HPV vaccine were their religious belief and culture; in particular, their belief that abstinence from sex before marriage would provide protection from disease [22,31,36]. Awareness of a health intervention is recognised as necessary but not sufficient condition for performing a health behaviour. As women become aware of HPV vaccine, they may have additional questions or concerns that may function as barriers to getting their daughters vaccinated [31] (Table 4).

Most studies were of generally good quality. When scored against the checklist used, ten qualitative studies received eight out of a possible 10 points, and one 10 of 10 [37]. Four of the eight quantitative observational studies scored eight of nine points, and the other scored seven of nine points (Table 5).

## 4. Discussion

This systematic review identifies gaps in knowledge, attitudes, and perceptions about HPV infection and its vaccine among immigrant parents in western countries. Our analyses indicate that although HPV vaccine has been in use for over a decade, information about this vaccine, and HPV infection in general, and its relation to cancer in particular, does not appear to have been well disseminated to immigrant parents. Most participants in 12 included studies had no knowledge about HPV vaccine (Table 2), one third of participants in two studies reported receiving no information about HPV vaccine, [27,35]. All participants in one study have not even heard of the vaccine [29]. This systematic review showed participants had both negative and positive attitudes towards HPV vaccination, and most participants had misconceptions about HPV vaccination.

In concordance with our systematic review findings, semi-structured interviews conducted with non-parent immigrant participants also showed limited knowledge about HPV infection its vaccine. For example, a study conducted in a Western Canadian province, found participants had limited knowledge about HPV. Most women perceived their risk of HPV to be low but reported willingness to receive the vaccine when recommended by their doctors [19]. Similarly [35], in Italy, knowledge and attitude toward HPV infection and vaccination among non-parent immigrants and refugees was low [40]. In Sweden, adolescent school students were interviewed in relation to their beliefs and knowledge about HPV prevention: HPV vaccination was found to be associated with ethnicity and the mothers’ education level; i.e., girls with a non-European background, including those of Arabic background, and with a less educated mother were less likely to have received the vaccine. Vaccinated girls perceived HPV infection as more severe, had more insight into women’s susceptibility to the infection, perceived more benefits of the vaccine as protection against cervical cancer and had a higher intention to engage in HPV-preventive behaviour [41].

Furthermore, another systematic review that explored knowledge and attitudes of Iranian people towards HPV vaccination found that the overall knowledge and awareness about HPV vaccination was low; however, their attitude toward HPV vaccination was positive and strong [42]. This corroborates the findings from three studies included in our systematic review that showed positive attitude towards HPV vaccines once parents were informed about it during focus groups. [34,36]. This could possibly explain why the negative attitude to HPV vaccination found in most of the studies included in our systematic review was stemmed from poor knowledge/misconceptions and may change after providing the right information.

Unlike the immigrants, mainstream populations of USA had better knowledge and more positive attitudes toward HPV vaccine. A quantitative study conducted in Southern California compared knowledge and acceptability between US-born African Americans and African immigrants, and between US-born Latinas and Latina immigrants. African and South American immigrants were less likely to know where they can get/refer for HPV vaccine and less likely to have heard about HPV vaccine than South Americans and US-born Africans [43]. Similarly, a study in Denmark found that refugee girls, mainly from Muslim countries, had significantly lower HPV immunization uptake compared to Danish born girls, indicating that refugee girls may face challenges to access and use of immunization services [44].

A study in 2018 indicated that the increase in refusal and hesitancy of Muslim parents to accept childhood vaccination was identified as one of the contributing factors in the increase of vaccine-preventable diseases cases in several countries such as Afghanistan, Malaysia and Pakistan. News disseminated via some social media outlets claiming that the vaccine has been designed to weaken Muslims, reinforced the suspicion and mistrust of vaccines by parents [45]. A qualitative study of the views of young non-parent Somali men and women in the USA demonstrated that the participants had limited knowledge about the vaccination and had suspicions concerning the effectiveness or value of immunization, with most participants stating that the Somali community was mostly Muslim and did not engage in sexual activity before marriage [46]. A cross-sectional study included in our systematic review conducted to evaluate awareness of women from major UK ethnic minority groups (Indian, Pakistani, Bangladeshi, Caribbean, African and Chinese women) toward HPV vaccination identified that those from non-Christian religions were less accepting of the vaccine (17–34%). The study concluded that some cultural barriers could be addressed by tailored information provided to ethnic minority groups [47].

Attitudes toward HPV vaccine are important in HPV vaccine uptake. Our systematic review revealed certain attitude-related barriers to vaccine acceptability for adolescents, particularly vaccine hesitancy among some mothers. A qualitative study reported that Latin American immigrant mothers of adolescent daughters expressed more hesitancy regarding adolescent vaccines compared to childhood vaccines expressed an increased sense of belief in their ability to determine what is best for their children [48]. In contrast to the negative attitudes of immigrant parents as found in most of the included studies in our systematic review, most mainstream non-immigrant women had positive attitudes about receiving an HPV vaccine and high intention to receive the vaccine both for themselves and their daughters [49]. Variables associated with intention to vaccinate included knowledge, personal beliefs, confidence that others would approve of vaccination, and having a higher number of sexual partners [49]. However, negative or variable attitudes of parents to vaccinate their children have been reported in a systematic review involving Turkish population [50]. The systematic review showed that between 14.4% and 68.0% of Turkish parents were willing to have their daughters vaccinated with HPV vaccine and between 11.0% and 62.0% parents were willing to have their sons vaccinated [50], suggesting a negative attitude may not be just a phenomenon of immigrants, many non-immigrants in their own countries too may have negative attitudes towards HPV vaccination. However, since this attitude appeared amenable to change in our systematic review, innovative simple interventions may improve attitudes to HPV vaccination. For instance, a higher vaccination rate was achieved at three clinics in Texas, USA among children and adolescents through the involvement of patient navigators. The patient navigators met the parents of unvaccinated or incompletely vaccinated children while they waited for their children’s health providers in private clinic rooms to confirm the need for additional HPV vaccine doses. Parents of children who needed ≥ 1 dose were offered personal counselling and given handouts in English or Spanish on HPV vaccine. Following such counselling about 67% parents got their children vaccinated either immediately or at a follow-up visit soon thereafter, indicating that providing counselling in a clinic setting can improve vaccination acceptance [51].

To our knowledge this is the first systematically conducted review of HPV vaccination knowledge, attitudes and perceptions among immigrants. Most included studies were of acceptable quality. We failed to identify research regarding knowledge, attitudes and perceptions of immigrant parents towards HPV vaccine in developing countries. Some papers did not clearly distinguish between attitudes and perceptions as outcomes. However, these studies suggest that tailored educational programs to improve KAP on HPV vaccine among immigrant parents may be a valuable intervention for HPV vaccination uptake.

## 5. Conclusions

Parental knowledge and attitudes towards HPV vaccine have been examined in many recent studies and lower uptake of HPV vaccine among immigrants, refugees and ethnic minorities has been documented. Our results support the pressing need to develop an intervention aimed to improve HPV vaccination uptake in these populations. More research is needed in the design and evaluation of tailored educational resources for ethnic minority groups, particularly in the framework of the vaccination programme.

## Figures and Tables

**Table 1:** Human papillomavirus (HPV) vaccination programs in several countries that receive high numbers of immigrants from developing countries.

Countries	Year Vaccination Introduced	Vaccination Strategy	Recent Reported Coverage (Year of Data) *	Immigrant’s Countries of Origin	HPV Vaccination in Countries of Origin
USA	2006	Primary care/health centre-based	48.6% (2017)	Mexico, China, Vietnam, South Korea, Portugal, Puerto Rico, Brazil, Argentina, Colombia, Peru, and other parts of South America; South Asia; Somalia, Ethiopia, Eritrea, and other African countries	Many countries of South America notably Mexico, Argentina, Brazil and Colombia have implemented national HPV vaccination, in the remaining countries it has not been implemented or still at preparatory stage
Denmark	2008	Primary care/health centre-based	40% (2018), but improving now	Poland, Syria, Turkey, Lebanon, Iraq, Palestine	In most of these countries there is no publicly funded national human papillomavirus vaccination programme
Netherlands	2010	Mixed: School-based and primary care-based	45.5% (2018)	European countries, Japan, USA, Australia, Indonesia, Turkey, Surinam, Morocco and Somalia	Except for the Western immigrants, most non-Western immigrants don’t have a vaccination policy in their home countries.
UK	2008	School-based	83.8% (2017/18)	Indian subcontinent, Poland, China, Nigeria, Somalia, Central America, and many other countries of Asia, Africa and Europe	In large majority of these countries there is no publicly funded national human papillomavirus vaccination programme but started in some countries (e.g., Sri Lanka) in recent years
Sweden	2011	School-based	80% (2018)	Middle East, Africa, Asia, Eastern Europe	In large majority of these countries there is no publicly funded national human papillomavirus vaccination
Puerto Rico	2006	Primary care/health centre-based	49.9% (2014)	Mainly from Dominican Republic	In Dominican Republic school-based HPV vaccination was announced in 2016

* If not specified this coverage data is for adolescent girls.

**Table 2:** Studies reporting knowledge of immigrants about HPV vaccine (16 articles).

Author(s), Publication Year [Ref]	Country of Study	Year of Study	Countries of Origin	Population	Mean Age in Years (Range)	Gender (n)		Knowledge Results
						Male	Female	
Aragones et al., 2016 [21]	New York City, USA	Not reported	Colombia, Dominican Republic, Ecuador, Mexico	36 Latino immigrants	42 (25–65)	3	33	Most parents were either not informed or possessed inaccurate knowledge about HPV and HPV vaccine.
Forster et al., 2016 [35]	Southwest England, UK	1 March 2015–1 March 2016	Indian subcontinent, Caribbean, Africa	33 Minority ethnic	47 (36–62)	1	32	Thirteen immigrant parents had not heard about HPV vaccine.
Glenn et al., 2015 [27]	Los Angeles, USA	January 2009–January 2010	Latina, China, Korea, Africa and others	490 Minorities ethnic	44 (7.2)		490	One third of participants had never heard of HPV or HPV vaccine and had low knowledge. About 63% (n=306) of respondents heard of HPV and another 61% (n=294) heard of HPV vaccine.
Kepka et al., 2015 [24]	Salt Lake City, USA	Not reported	Mexico, Puerto Rico, Brazil, Argentina, Peru, and Portugal	118 Mexican immigrants	18–50 (±2.4)	18	97	Majority had no knowledge about HPV vaccine.
Mupandawana1 et al., 2016 [38]	North England, UK	Not reported	South Africa, Zimbabwe, Nigeria, Kenya, and Zambia	10 African immigrants	Not reported	5	5	Most participants had inaccurate knowledge about HPV vaccine.
Allen et al., 2012 [30]	Boston, USA	February -May 2008	Hispanic and African American	64 immigrants	Not reported	19	45	The majority of parents felt that they did not have adequate information about HPV or HPV vaccine to make an informed decision.
Salad et al., 2015 [29]	Netherlands	March–June 2013	Somalia	6 immigrants	(23–66)		6	Participants described a lack of information about HPV vaccine.
Luque et al., 2012 [26]	Georgia, USA	Not reported	Mexico and Honduras	12 Hispanic immigrants	(25–44)	7	5	Parents had little knowledge about HPV vaccine.
Bodson et al., 2016 [25]	Salt Lake City, Utah, USA	August 2013–October 2013	Mexico and others	108 Hispanic/Latino immigrants101 born out USA	(16– >50)	16	92	Participants born in Mexico or elsewhere (Spanish background) had lower factual knowledge than participants who were born in the United States. In total, 67.3% of participants had heard of HPV vaccine and 76.4% of HPV.
Marlow et al., 2009 [39]	UK	July–August 2008	Indian subcontinent, Caribbean, Africa, China	Ethnic minority	(16– >50)		601	Almost half of ethnic minority parents had not heard of the vaccine before being invited to vaccinate their daughters.
Greenfield, et al., 2015 [23]	Washington, USAWashington, USA	Not reported	Mexico, Somalia, Ethiopia and Eritrea	156 immigrants’ parents	41		Not reported	Lack of knowledge about HPV vaccine was the main reason given by parents that their adolescents had not been vaccinated.
Zeraiq et al., 2105 [37]	Denmark	January 2011–January 2012	Lebanon, Iraq, Palestine	23 immigrants	Not reported		23	Ethnic minorities had lack of knowledge about HPV and HPV vaccine.
Grandahletal., 2012 [36]	Uppsala, Sweden	February–June 2011	Middle East, Africa, Asia, East ern Europe	50 immigrants	(18 [28]–60)		50	The participants had limited knowledge about HPV and cervical cancer and HPV vaccine. Lack of knowledge was the main reported barrier to vaccination.
Hopfer et al., 2017 [32]	CA, USA	July 21–August 20, 2016.	Latina and Vietnamese	48 immigrants	(18–26)		48	Lack of awareness about HPV was evident in women’s stories, including confusing HPV with HIV, not knowing that HPV is a sexually transmitted infection. Vietnamese participants (96% (23/24)) were unable to elaborate on what HPV was, many were uncertain about its significance, 25% (2/8) unvaccinated Latina had never heard of HPV.
Stephens et al., 2014 [22]	Haiti, USA	October 2010–May 2011	Haiti	31 immigrants.	(18–22 yrs.)		31	Mothers had no knowledge about HPV (80.6% (25/31)), very knowledgeable (3.2% (1/31)), fairly knowledgeable (12.9% (4/31)), somewhat knowledgeable (3.2% (1/31)). Mothers had no knowledge about HPV vaccine (83.9% (26/31)), very knowledgeable, fairly knowledgeable (9.7% (3/31)), somewhat knowledgeable (6.4% (2/31)).
López, et al., 2016 [33]	San Juan, Puerto Rico	Not reported	Dominican Republic	60 immigrants	38.6 (± 7.2 yrs.)	5	55	Parents had not heard about HPV (3.3% (2/60)) and yes heard (91.7% (55/60)). Parents had not heard about HPV vaccine for males (38.3% (23/60)), had heard (55% (33/60))

**Table 3:** Studies reporting attitudes of immigrants about HPV vaccine (13 articles).

Author(s), Publication Year [Ref]	Country of Study	Year of Study	Population	Mean Rge in Years (Range)	Gender (n)		Attitudes Results
					Male	Female	
Aragones et al. 2016 [21]	NYC (New York City), USA	Not reported	36 immigrants	42 (25–65)	3	33	Parents were motivated to protect the health of their children and were keen to obtain more information regarding HPV and the vaccine.
Forster et al., 2017 [35]	Southwest England	1 March 2015–1 March 2016	33 Ethnic minorities	47 (25–65)	1	32	Ethnic minority mothers said HPV vaccine was unnecessary as they had been fine without it. Parents expressed a wide range of concerns about the vaccine. A number of non-vaccinating ethnic minority parents believed their daughters were not at risk of contracting HPV or developing cervical cancer.
Glenn et al., 2015 [27]	Los Angeles, USA	January 2009–January 2010	Ethnic minorities	44 (7.2)		490	Ethnic minorities had positive and negative attitudes towards HPV vaccine: 63% of participants expressed positive attitudes towards immunization against HPV disease is a good thing. Participants with negative attitudes (54%): that Immunizations have more side effects than benefits.
Albrigh et al., 2017 [28]	Colorado, USA	July 2012–January 2013	41 Ethnic minorities	(18– >50)	3	38	The most common reported reasons for non-initiation and non-completion among English-speaking parents included a low perceived risk of HPV infection, vaccine safety concerns, and distrust of government and/or medicine. Spanish-speaking parents who had either not encouraged initiation of HPV vaccine series or had not explained the necessity of completing the series, cited concerns that vaccination would encourage sexual activity.
Mupandawana et al., 2016 [38]	North England, UK	Not reported	10 Ethnic minority	Not reported	5	5	Majority of participants said HPV vaccine was unacceptable, with fear of promiscuity, infertility and concerns about it being a new vaccine with unknown side effects. Religious values and cultural norms influenced vaccine decision-making with fathers acting as the ultimate decision-maker.
Allen et al., 2012 [30]	Boston, USA	February–May, 2008	64 Ethnic minority	Not reported	19	45	Participants distrust medical providers and pharmaceutical companies.
Salad et al., 2015 [29]	Netherland	March to June 2013	6 Immigrants	(23–66)		6	Most mothers have distrust towards the Dutch health care system and government and doubts about HPV vaccine age.
Marlow et al., 2009 [39]	UK	July to August 2008.	Ethnic minority	(16– >50)		601	Parents with strong religious or cultural views were less likely to accept HPV vaccine. Consistency with attitudes to HPV testing, which some minority women felt reflected non-traditional cultural or religious practices and were concerned it encouraged premature sex.
Greenfield et al. 2015 [23]	Washington, USA	Not reported	156 immigrants	41		156 gender not distinguished	All three ethnic groups expressed a desire to access vaccine information in their respective languages.
Zeraiq et al., 2015 [37]	Denmark	January 2011 to January 2012	23 Ethnic minority	Not reported		23	All participating mothers accepted the vaccine for their daughters to prevent cervical cancer.
Grandahl et al., 2012 [36]	Uppsala, Sweden	February to June 2011	50 immigrants	(18–60)		50	Participants’ expressed that they accepted the vaccination for their daughters, as it was important for their future health. Some women considered girls in the target group were too young and it would be better to wait until they were a little older and had become women.
Perkins et al., 2010 [34]	Boston, Massachusetts, USA	June 2007 to February 2008.	72 Immigrants	Not reported	3	69	Attitudes differed dramatically by ethnicity; only 11% of Caucasian parents endorsed school HPV vaccine entry requirements, compared with 78% of African-American parents, 60% of Afro-Caribbean and African parents, and 90% of Latino parents. Most parents expressed favorable opinions toward HPV vaccine for their own daughters.
Stephens et al., 2014 [22]	Haiti, USA	October 2010–May 2011	31 immigrants	(18–22)		31	Immigrant mothers who had little knowledge about HPV or the vaccine, felt unsure about vaccination; their concern centered on conflict with cultural values and perceptions of risks associated with HPV vaccine.

**Table 4:** Studies reporting perceptions of immigrants about HPV vaccine (12 articles).

Author(s), Publication Year [Ref]	Country of Study	Year of Study	Population	Age	Gender (n)		Perception Results
					Male	Female	
Forster et al., 2016 [35]	southwest, England	1 March 2015−1 March 2016	33 Ethnic minority	47 (25–65)	1	32	Non-vaccinating ethnic minority parents reassured themselves of their decision by reporting that there are approaches other than vaccination to protect against HPV, such as abstinence from sex before marriage, which was related to religious beliefs.
Mupandawana1 et al., 2016 [38]	North England, UK	Not reported	10 Ethnic minority	Not reported	5	5	HPV vaccine was generally unacceptable within this African community, with culture and religion influencing risk perceptions toward the vaccine and playing important roles in vaccination decision making.
Salad et al., 2015 [29]	Netherland	March–June 2013.	6 Immigrants	(23–66)		6	Participants’ belief that abstinence from sex before marriage protect from diseases.
Luque t al., 2012 [26]	Georgia, USA	Not reported	Hispanic immigrant	Not reported	7	5	Participants had misperceptions about HPV vaccine. They think that the vaccine is unnecessary if they are not having sex.
Albright et al., 2017 [28]	Colorado, USA	July 2012–January 2013	41 Ethnic minority	(18 to 50)	3	38	Spanish-speaking parents concerned that vaccinating against HPV would encourage sex. These parents expected their daughters to abstain from sex until marriage, and they did not want to give their daughters the message that sexual activity was permissible or give them a false protection.
Marlow et al., 2009 [39]	UK	July–August 2008.	950 Ethnic minority	(16– >50)		601	The main reason for declining HPV vaccine was religious belief. The importance of religion appears to come from a strong belief in sexual abstinence until marriage.
Greenfield et al. 2015 [23]	Washington, USA	Not reported	156 Immigrants	41		156 gender not stated	All three minority ethnicities had misperceptions about HPV vaccine or HPV disease. Most participants do not believe children are at risk and believe the vaccine could lead to early initiation of sexual activity.
Grandahl et al., 2012 [36]	Uppsala, Sweden	February–June 2011	50 immigrants	(18–60)		50	Cultural influences on perceptions about protection: participants believed a woman did not have sexual intercourse with a man before marriage.
Baldwin et al., 2012 [31]	Texas, USA	December 2008–May 2010	256 Ethnic minority	42.3		256	Non-White participants were significantly less likely to have talked with others and looked for information about HPV vaccine than White participants. Mothers’ perceptions of vulnerability, severity, varied by race/ethnicity.
Stephens et al., 2014 [22]	Haiti, USA	October 2010–May 2011.	31 Immigrants	(18–22)		50	Most mothers were willing to have their daughters vaccinated against HPV if it would protect or improve their health. Some mothers did not support HPV vaccine for their daughters; the remaining mothers were unsure because of their lack of knowledge. For those mothers who were unsure; concerns centred on conflict with cultural values and their perceptions of the risks associated with the vaccine.

**Table 5:** Quality assessment of the included studies.

Author (Ref)	Score	Remarks
**Qualitative Studies Assessed by Joanna Briggs Institute Critical Appraisal Checklist**		
Aragones et al., 2016 [21]	Met 8 of 10 positive criteria	
Allen et al., 2012 [30]	Met 10of 10 positive criteria	Nil
Zeraiq et al., 2105 [37]	Met 10 of 10 positive criteria	
Albright et al., 2017 [28]	Met 8 of 10 positive criteria	
Grandahl et al., 2012 [36]	Met 8 of 10 positive criteria	
Stephens et al., 2014 [22]	Met 8 of 10 positive criteria	
Forster et al., 2016 [35]	Met 8 of 10 positive criteria	
Mupandawana et al., 2016 [38]	Met 8 of 10 positive criteria	
Salad et al., 2015 [29]	Met 7 of 10 positive criteria	
Luque et al., 2012 [26]	Met 8 of 10 positive criteria	
Perkins et al., 2010 [34]	Met 8 of 10 positive criteria	
Hopfer et al. 2017 [32]	Met 8 of 10 positive criteria	
**Quantitative Studies Assessed by Newcastle Ottawa Scale**		
Baldwin et al., 2012 [31]	Scored 7 of 9 stars	
Bodson et al., 2016 [25]	Scored 7 of 9 stars	It is a mixed method study
Glenn et al., 2015 [27]	Scored 7 of 9 stars	Nil
Greenfield et al., 2015 [23]	Scored7 of 9 stars	It is a mixed method study
Kepka et al., 2015 [24]	Scored 7 of 9 stars	Nil
López, et al., 2016 [33]	Scored 8 of 9 stars	
Marlow et al., 2009 [39]	Scored 8 of 9 stars	
